# 1154. Safety and Efficacy of Ceftolozane/Tazobactam Plus Metronidazole Versus Meropenem in Pediatric Participants With Complicated Intra-abdominal Infection: A Phase 2, Randomized Clinical Trial

**DOI:** 10.1093/ofid/ofab466.1347

**Published:** 2021-12-04

**Authors:** Carl-Christian A Jackson, Jason Newland, Natalia Dementieva, Julia Lonchar, Feng-Hsiu Su, Jennifer A Huntington, Mekki Bensaci, Myra W Popejoy, Matthew G Johnson, Carisa S De Anda, Elizabeth G Rhee, Christopher Bruno

**Affiliations:** 1 Tufts Children’s Hospital, Boston, Massachusetts; 2 Washington University, St. Louis, Missouri; 3 Regional Children’s Hospital, Dnipropetrovsk, Dnipropetrovs’ka Oblast’, Ukraine; 4 Merck & Co., Inc., Kenilworth, New Jersey

## Abstract

**Background:**

Ceftolozane/tazobactam (C/T), a cephalosporin–β-lactamase inhibitor combination, is approved for treatment of complicated urinary tract infections, complicated intra-abdominal infections (cIAI), and nosocomial pneumonia in adults. Safety and efficacy of C/T in pediatric participants with cIAI was assessed.

**Methods:**

This phase 2 study (NCT03217136) compared C/T + metronidazole (MTZ) with meropenem (MEM) for treatment of cIAI. Age- and weight-adjusted dosing is summarized in Table 1. The primary objective was to evaluate the safety and tolerability of C/T + MTZ compared with MEM. A key secondary endpoint was clinical cure at end of treatment (EOT) and test of cure (TOC).

Table 1. Summary of Dosing and Pharmacokinetic Sampling Schedule by Age Cohort

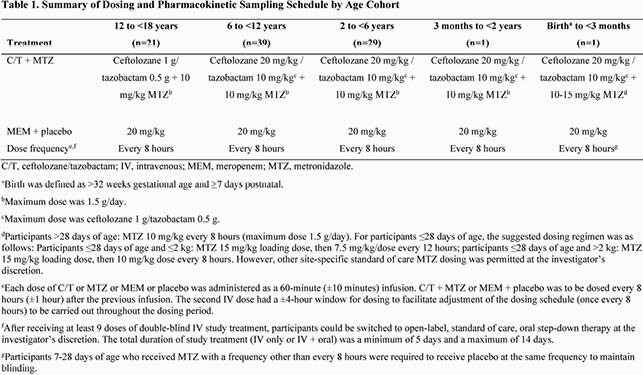

**Results:**

A total of 94 participants were randomized 3:1; 91 were treated with C/T + MTZ (n=70) or MEM (n=21) comprising the modified intent-to-treat (MITT) population. The clinically evaluable population included 78 participants at EOT (C/T + MTZ, n=59; MEM, n=19) and 77 participants at TOC (C/T + MTZ, n=58; MEM, n=19). The most common diagnosis and pathogen in the MITT population were complicated appendicitis (C/T + MTZ, 91.4%; MEM, 100%) and Escherichia coli (C/T + MTZ, 67.1%; MEM, 61.9%). The mean (SD) intravenous therapy/overall treatment duration was 6.4 (2.8)/9.3 (3.6) days and 5.8 (1.8)/9.0 (3.2) days for C/T + MTZ and MEM, respectively. In total, ≥1 adverse events (AE) occurred in 80.0% and 61.9% of participants receiving C/T + MTZ and MEM, respectively (Table 2), of which 18.6% and 14.3% were considered drug related. Serious AE occurred in 11.4% (8/70) and 0% (0/21) of participants receiving C/T + MTZ and MEM, respectively; none were considered drug related. No drug-related study drug discontinuations occurred. In the MITT population, rates of clinical cure for C/T + MTZ and MEM at EOT were 80.0% and 95.2%, and at TOC were 80.0% and 100%, respectively (Figure 1); 6 of the 14 failures for C/T + MTZ were indeterminate responses scored as endpoint failures per protocol. In the clinically evaluable (CE) population, rates of clinical cure for C/T + MTZ and MEM were 89.8% and 100% at EOT, and 89.7% and 100% at TOC, respectively (Figure 1).

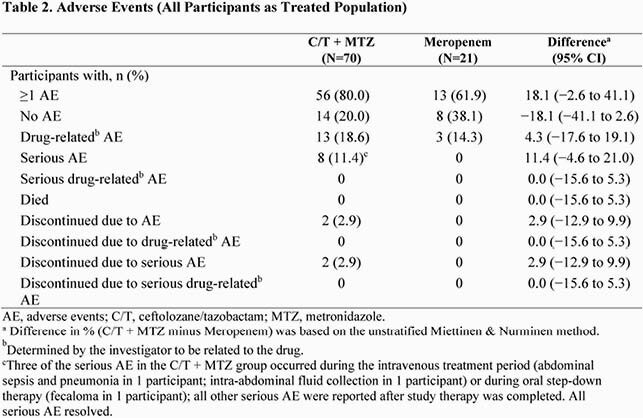

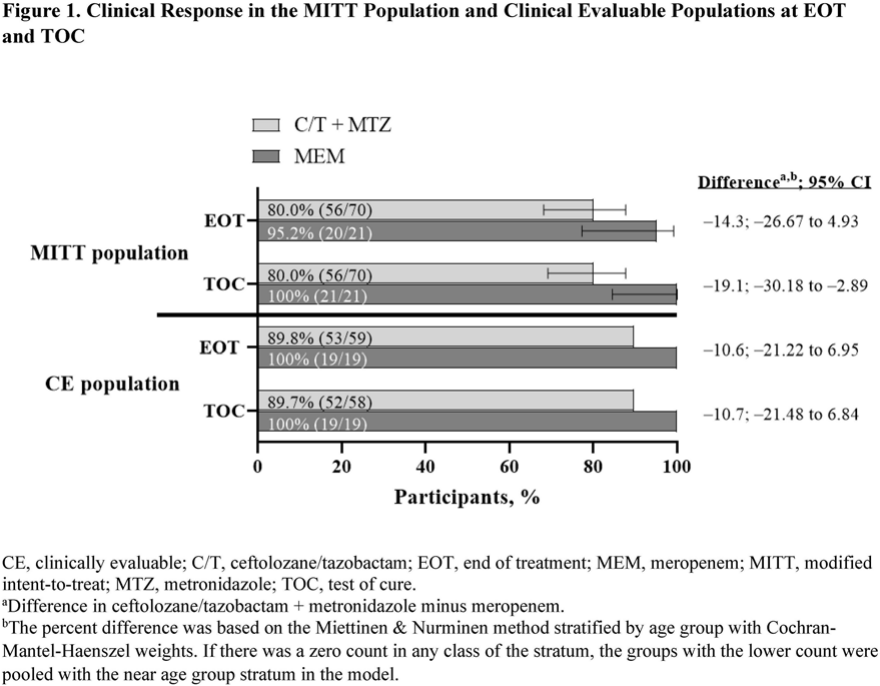

**Conclusion:**

C/T + MTZ was well tolerated in pediatric participants with cIAI, and rates of clinical success were high with C/T treatment. C/T is a promising new treatment option for children with cIAI.

**Disclosures:**

**Carl-Christian A. Jackson, MD**, **Merck & Co. Inc.** (Shareholder) **Julia Lonchar, MSc**, **Merck Sharp & Dohme Corp.** (Employee, Shareholder) **Feng-Hsiu Su, MPH, MBA**, **Merck Sharp & Dohme Corp.** (Employee, Shareholder) **Jennifer A. Huntington, PharmD**, **Merck Sharp & Dohme Corp., a subsidiary of Merck & Co., Inc., Kenilworth, NJ, USA** (Employee) **Mekki Bensaci, PhD**, **Merck Sharp & Dohme Corp., a subsidiary of Merck & Co., Inc., Kenilworth, NJ, USA** (Employee) **Myra W. Popejoy, PharmD**, **Merck Sharp & Dohme Corp.** (Employee) **Matthew G. Johnson, MD**, **Merck Sharp & Dohme Corp., a subsidiary of Merck & Co., Inc., Kenilworth, NJ, USA** (Employee) **Carisa S. De Anda, PharmD**, **Merck Sharp & Dohme Corp.** (Employee, Shareholder) **Elizabeth G. Rhee, MD**, **Merck Sharp & Dohme Corp** (Employee, Shareholder) **Christopher Bruno, MD**, **Merck Sharp & Dohme Corp., a subsidiary of Merck & Co., Inc., Kenilworth, NJ, USA** (Employee)

